# Evaluation of the child growth monitoring programme in two Zimbabwean provinces

**DOI:** 10.4102/phcfm.v14i1.3373

**Published:** 2022-07-06

**Authors:** Anesu Marume, Saajida Mahomed, Moherndran Archary

**Affiliations:** 1 School of Nursing and Public Health, College of Health Sciences, University of KwaZulu-Natal, Durban, South Africa; 2 Ministry of Health and Child Care Zimbabwe, Health Promotion, Government of Zimbabwe, Harare, Zimbabwe; 3 Department of Paediatrics, College of Health Sciences, University of KwaZulu-Natal, Durban, South Africa

**Keywords:** adverse nutrition outcome, children, weight-for-age, height-for-age, growth faltering, growth monitoring, Zimbabwe

## Abstract

**Background:**

The child growth monitoring (CGM) programme is an important element of nutrition programmes, and when combined with other child health programmes, it can assist in successful management and control of malnutrition in children.

**Aim:**

This study aimed to assess the extent to which the CGM programme is able to identify instances of childhood malnutrition and how much this contributes towards malnutrition reduction in Zimbabwe.

**Setting:**

The study was conducted in Manicaland and Matabeleland South provinces of Zimbabwe. The two provinces were purposively selected for having the highest and least proportion of children affected by stunting in the country.

**Methods:**

The CGM programme in Zimbabwe was evaluated using the logic model to assess the ability of the programme to identify growth faltering and link children to appropriate care.

**Results:**

Records from 60 health facilities were reviewed. Interviews were conducted with 60 nurses, 100 village health workers (VHWs) and 850 caregivers (300 health facility exit interviews, 450 community based). Nearly all (92%) health facilities visited had functional measuring scales. Twelve health facilities (20%) had no functional height board, with five using warped height boards for measuring children’s height. Less than a quarter (21%) of the children had complete records for weight for age and height for age. A large proportion of children eligible for admission for the management of moderate (83%) and severe malnutrition (84%) were missed.

**Conclusion:**

The CGM programme in Zimbabwe is not well equipped for assessing child height for age and management of children identified with malnutrition, thus failing to timely identify and manage childhood stunting.

## Introduction

Adverse nutrition outcomes can have a significant impact on the economic landscape of a community and nations.^1,2,3^ Evidence has shown that adverse nutrition outcomes can impact a child’s mental capacity, immunity and social skills in later stages of life.^4,5,6,7^ More than 140 million children below five years of age globally are stunted, and nearly 50 million are wasted.^8^ In order to reverse the malnutrition tide, timely identification and management of growth faltering are required.^9^ A growth monitoring programme is designed to identify children with nutritional complications and institute remedies timeously.^10,11^

Although nearly all countries globally have growth monitoring programmes,^12,13,14^ there is conflicting evidence on the effectiveness of these programmes. Many studies have shown that health facility-based growth monitoring programmes rarely result in reducing negative nutrition outcomes.^14,15^ However, evaluations of community growth monitoring programmes have shown that it is highly effective in both reducing negative nutrition outcomes and increasing knowledge levels of mothers regarding the nutritional needs of their children.^16,17,18,19^ The child growth monitoring (CGM) programme is an important element of nutrition programmes, and when combined with other child health programmes, it can assist in successful management and control of malnutrition in children. Two community trials found educating mothers on how to read the child health card as effective in improving child nutrition outcomes.^20^ A comprehensive review of the literature found conflicting evidence on the impact of CGM programme. The review identified a poor linkage with other services as a weakness of growth monitoring programmes as often health workers would weigh children but would not provide counselling or ways of reversing identified adverse nutrition outcomes.^21^ While coming up with mixed conclusions on the impact of the growth monitoring programme, the same review highlighted the strength of growth monitoring in several areas that include early detection of growth faltering, increased contact with health services, improved maternal knowledge, access to other services, reduced morbidity and improved nutritional status.^21^

In Zimbabwe, the CGM programme is responsible for identifying children with nutrition deficiencies and tracks children’s growth and development from birth to five years. Children with nutritional deficiencies are identified and referred for further management to the Integrated Management of Acute Malnutrition (IMAM) programme (wasting, underweight and overweight) and the Child Feeding Programme (stunting, wasting, underweight and overweight). The CGM programme starts at the community level led by village health workers (VHWs). Nurses at the health facilities within the catchment area of the VHW were responsible for training, monitoring and supervising activities conducted by the VHWs. In some districts, household-based growth monitoring is being piloted to strengthen the uptake of growth monitoring activities. In the household growth monitoring programme, families with new-born children are being provided with mid upper arm circumference (MUAC) tapes and height meters. The health facility-based growth monitoring is carried out at primary health clinics where moderately malnourished children are admitted into the Outpatient Therapeutic Programme (OTP) and provided with ready-to-use therapeutic food (RUTF) sachets depending on the stage of malnutrition. Moderately malnourished children who fail to recover in the OTP and severely malnourished children are admitted into the Stabilisation Care (SC) Programme at the hospital level.

For growth monitoring programmes to be a success, it must be accessible to the targeted population. A desk review of the growth monitoring programme at both community and health facility levels in rural Zimbabwe reported that less than 50.0% of children were accessing the growth monitoring programme.^15^ According to the National Nutrition Survey of 2018, Zimbabwe has a stunting prevalence of 26.2%, while the wasting prevalence increased from 2.0% in 2010 to 2.5% in 2018.^22^ The high stunting burden makes the need for timely identification of children with adverse growth outcomes critical to reverse its impact on the growing child.

### Study aim

This study aims to assess the ability of the CGM programme in identifying and initiating corrective measures against childhood malnutrition in Zimbabwe through a systematic evaluation of the growth monitoring and promotion programme in the provinces most and least affected by childhood stunting (Manicaland and Matabeleland South) in Zimbabwe

### Logic framework for growth monitoring

**FIGURE 1:**
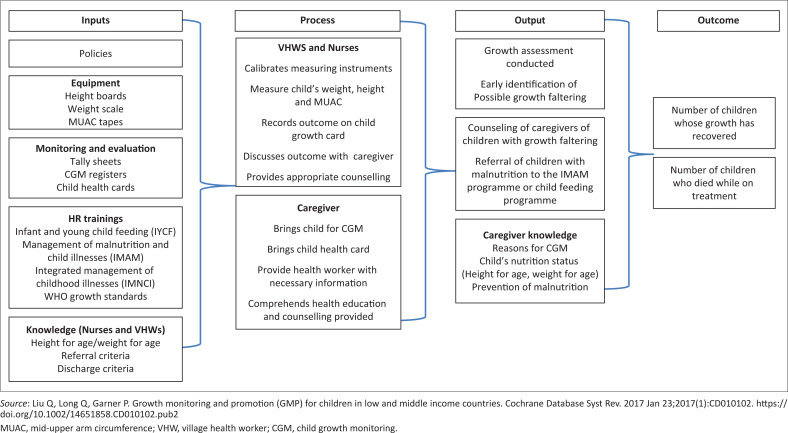
Logic model for growth monitoring.

This CGM programme was evaluated using the logic model.^20,23^ The logic model allows for the identification of strengths and weaknesses across the spectrum of the inputs, processes, outputs and outcomes of the CGM programme (Figure 1).^20^

## Materials and methods

### Study setting

The study was conducted in Manicaland and Matabeleland South provinces in Zimbabwe. The two provinces have the least (Matabalelend South province) and second highest (Manicaland Province) populations in Zimbabwe.

### Study variables

Using the logic model, we looked at inputs, processes output and outcome indicators for the CGM programme in Zimbabwe (Figure 1). With regard to trainings, four out of a possible ten trainings on child health were considered for this evaluation because of them being nationwide training and were predominantly focusing on CGM, management and treatment. These trainings were carried out on Infant and Young Child Feeding (IYCF), Integrated Management of Neonatal and Childhood Illnesses (IMNCI), World Health Organization (WHO) growth standards and IMAM.

### Study population and sampling

In order to ensure that data were representative of the two provinces, random selection of health facilities was done. A list of all health facilities (269 Manicaland Province, 161 Matabeleland South province) was developed; each health facility was assigned a number. The Microsoft Excel package was used to generate random numbers, which were then used to identify 30 health facilities per province. The sample size was calculated using the formula for proportions, differences from constant at a power of 0.95, error of 0.05, and effect size of 0.3 for health facility selection, 0.2 for VHW selection and 0.1 for caregiver selection. The differences in effect size were advised by the assumption that the source population increased as we move from nurses to caregivers. The calculation after rounding to the nearest 10 estimated interviewing 60 nurses, 100 VHWs and 300 caregivers. The list of all the VHWs (2500) operating within the 60 selected health facilities was created. Random numbers was also used to generate a sample of 50 VHWs per province for interviewing. The 300 caregivers were conveniently selected for observations and exit interviews. Thus, five mothers or caregivers, who had visited the health facility for CGM, were purposively selected at each of the selected health facilities. At each of the health facility, the nurse-in-charge (senior nurse at primary clinic level or senior nurse in a select ward at hospital level) was interviewed. If the nurse-in-charge was absent or did not have experience working in the growth monitoring programme, a representative was interviewed. To avoid selection bias caused by interviewing caregivers identified at health facility only, an additional sample of 450 caregivers with children aged 6–59 months was recruited from the community within the catchment area of the health facilities. Caregivers of the 450 children were interviewed, while their children’s health cards were reviewed for completeness.

### Data collection process

Three BSc Nutrition students were recruited and trained on the data collection techniques. The students would collect data at each health facility as a team before proceeding to the next health facility. Data were collected between June 2020 and August 2020 through observations, interviews and reviews of growth monitoring records. Interviews were carried out using an android-based data collection tool. Interviews were conducted to assess the inputs available at the health facility and community level, growth monitoring processes and knowledge levels of both health workers and caregivers regarding the growth monitoring programme. The number of growth monitoring sessions each child was exposed to and the possible reasons for missing any session were sought through caregiver interviews in Shona, Ndebele or English according to the interviewee preference. The use of a checklist was observed in notebooks and transcribed by the research team. Observations were conducted at each of the selected health facilities and included the techniques for measuring weight, height and MUAC; the consultation; and counselling process, and if there was any referral for children who required additional assistance. The records reviewed included the child health card, the health facility growth monitoring register, the health facility asset register, the VHW growth monitoring register, district nutrition reports, and the health facility malnutrition records and reports. Data collected from these reports included the number of children weighed, had their height measured, had their MUAC measurement recorded and the number of children referred for further management. Data from all health facilities were available through the Ministry of Health and Child Care Health Information System (DHIS2), district mentorship reports and monthly tally sheet reports sent to the Health Information Office. Multiple data sources at the health facility and district level were analysed to ensure the accuracy of the data. The district nutrition reports were considered validated as these reports undergo data quality assessments. The child anthropometric data reported and analysed were based on the WHO growth standards.

### Data analysis

Data from the interviews were analysed using Stata 14.0 (StataCorp, College Station, TX, United States [US]). Frequencies, means and standard deviations were used to summarise sample characteristics. Qualitative data were organised in themes, and frequencies of responses were measured.

### Ethical considerations

Confidentiality was maintained throughout the data collection process, with no personal identification data collected. A written informed consent was sought from all interviewees before data collection. The Medical Research Council of Zimbabwe and the University of KwaZulu-Natal, Biomedical Research and Ethics Committee provided ethical clearance (BE109/19). The district medical officers provided gatekeeper approval.

## Results

### Inputs

All the districts within the two provinces had at least one facility running the SC programme, designated for the treatment and management of children with severe forms of malnutrition.

#### Equipment and reference materials

Of the 60 health facilities visited, 55 (92%) had functional measuring scales. However, most 36 (60%) of those facilities were relying on only one measuring scale. Of the five facilities that were using malfunctioning scales to assess weight, one of these scales could only display numbers when exposed to heat, and the health facility would use the scale under direct sunlight or next to a heater. Only six (10%) of the 60 health facilities visited were observed calibrating the measuring scales at the start of the working day, with no documentation of the calibration done. In interviews, the majority of the health workers stated that the measuring scales do not need to be calibrated daily with 48% saying once per quarter, 30% once a month and 11% once a week. The majority of health facilities had MUAC tapes 53 (88%) and height boards 48 (80%) for children. Most height boards at the clinic were properly stored except at three health facilities where the height boards were at the open waiting area. Five (8%) health facilities had visibly warped height boards. All health facilities had reference materials to use for growth monitoring, with the most common being the *WHO Growth Standards Handbook*. More than one-third (35%) of facilities (nine in Manicaland and 12 in Matabeleland South) had no stock of RUTF sachets.

One hundred VHWs were interviewed, with their mean age being 45 years (standard deviation [s.d.]: 10.6). A majority (89%) of them had reached the secondary level of education, and 75% were female children. Each VHW had an average of 49 children (s.d.: 29.3) below five years within their catchment area. Nearly, all VHWs had MUAC tapes for children (94%) and measuring scales (92%). However, only 6% of the VHWs stated that they had a height board, while a further 6% utilised improvised height gauges in the form of graduations on their walls or graduated wooden planks for measuring children’s height. A large proportion of VHWs (56%) stated that they measured the height of children in their catchment area at their reporting health facility. A quarter of the VHWs interviewed (26%) stated that they did not assess height measurements. A large proportion (64%) stated that they had reference materials to use while conducting the CGM programme. The most common reference materials were the *IYCF Handbooks* (30%), followed by the *WHO Growth Standards Handbook* (25%).

#### Training and knowledge of nurses and village health worker

Of the 60 nurses interviewed, 49 (82%) reported that they had been trained in IYCF. Less than half (47%) reported having been trained in WHO growth standards, 24 (40%) in IMAM and 19 (32%) in IMNCI. Overall, the nurses interviewed had an adequate knowledge of all the indicators of the CGM programme. However, knowledge on the referral and discharge of children from the OTP was low. Only six (10%) nurses were able to accurately describe the criteria for enrolling a child into the OTP, and only two (3%) knew the discharge criteria for children admitted into the OTP. Only two of the nurses (3%) were able to state the admission criteria into the SC programme, and none could accurately describe the discharge criteria from this programme.

Almost all the VHWs (98%) reported having been trained in Community Infant and Young Child Feeding, while 87% had received training in Community Management and Referral of Childhood Malnutrition. The majority of the VHWs (84%) interviewed accurately described the process for measuring the length of a child who is not yet able to stand, and 16%) could not accurately identify an underweight child using the child health card. Nearly, a quarter (24%) of the VHWs interviewed could not define stunting, while 20% were not able to identify a stunted child using the child health card. A further 27% did not know the effects of stunting on a growing child. Fifteen percent did not know why MUAC is measured, and 22% were not able to check for bilateral pitting oedema. All the VHWs could define a balanced diet and the importance thereof, and how a family can consume a balanced diet using local food sources.

### Process

#### Growth monitoring assessments

A secondary analysis of health facility CGM data showed no differences in the uptake of the CGM programme between Manicaland and Matabeleland South provinces. There were similar proportions of children who were assessed for weight (Manicaland 34%, Matabeleland South 33%) and MUAC (Manicaland 32%, Matabeleland South 32%) in 2019. However, only 25% and 22% of children had their height measured in Manicaland and Matabeleland South, respectively, in the same period.

Out of the 450 randomly selected caregivers, only seven (1.5%) did not have their child health card. A majority of the children 279 (62%) were weighted in the community, while a majority (95%) had their height measured at the health facility. Despite 88% (397) of the caregivers reporting that their children had both their height and weight measurements assessed, a high proportion of the child health cards had not been completed (height for age: 342 [77%], and weight for age: 245 [55%]).

A total of 279 out of a targeted 300 observations of interactions between a nurse and caregiver were conducted at 30 health facilities. Children’s height and MUAC measurements were carried out by the nurse, while weight measurements were carried out by multiple cadres (e.g. nurse aide, ward nutrition coordinator or a non-skilled volunteer, most commonly a VHW) before the client is seen by the nurse. In a majority of the observations, 85 (66%), the nurse explained the outcome of the growth monitoring assessment with guidance from graphs on the child health card. All children observed by research team had their weight measured, while 240 (86%) had their height measured and 187 (67%) had their MUAC assessed. The reasons for not measuring the height or MUAC were malfunctioning height board (4%), absence of height board (8%), unreadable MUAC tape (14%), no MUAC tape (9%) and health worker forgetting or missing one or more assessments (height 1%, MUAC 8%).

Four percent, 13% and 81% of the VHWs reported not measuring children’s MUAC, weight and height in 2019, respectively. Four percent, 22% and 20% of the VHWs had records for height, weight and MUAC, respectively, to indicate that they reached all the children in 2019 within their catchment area. The data from the VHW monthly return forms and the health facility monthly return forms indicated that in 2019, 25%, 20% and 5% of children are assessed for weight, MUAC and height, respectively, at the community level. Nearly, a third (32%) of the 3986 children in the interviewed VHW’s registers had no record of having had their anthropometric measurements taken. For the children who had their anthropometric data recorded in the VHW register, nearly 89% had at least six months of missing data.

#### Counselling and health education

A majority of the caregivers interviewed, 352 (78%), stated that the health workers explained the child measurements during child growth consultations. Nearly, three-quarters, 324 (72%), of the caregivers stated that they received health education when they visited the health facility for growth monitoring. The five most common health education topics provided include breastfeeding 311 (69%); appropriate infant feeding 282 (63%); family planning 267 (59%); water, sanitation and hygiene 196 (44%); and maternal nutrition 190 (43%). A majority of the caregivers, 310 (68%), stated that most of the health education on child growth was delivered by VHWs.

Village health workers who were interviewed stated that the most common platforms for providing health education on CGM, IYCF and management of malnutrition were mother or caregiver support groups (85%), formal community meetings (69%), door-to-door (53%) and funerals (25%). All nurses interviewed stated that they provided education on nutritional topics, such as exclusive breastfeeding, complementary and supplementary feeding while strengthening the importance of regularly taking their child for CGM.

### Outputs

#### Caregiver knowledge

A majority of caregivers, 329 (73%), interviewed were able to clearly describe the weight-for-age growth of their child using the child’s child growth curve. However, only 58% could clearly describe the height-for-age growth trajectory of their child. Furthermore, only 21% could accurately state the current height-for-age classification of their child. However, a large proportion, 351 (78%), could state the current weight-for-age classification of their child.

#### Screening and management of malnutrition

The secondary data analysis of health facility and community growth monitoring registers showed that 34%, 31% and 23% of children had their weight-for-age, MUAC and height-for-age measurements done. An analysis of 2019 data in the Integrated Management of Nutrition health facility registers showed that 83% of children who were eligible for admission into OTP (Manicaland 80%, Matabeleland South 89%) were not admitted. Another similarly large proportion of eligible children (84%) were also not admitted in the SC programme (Manicaland 83%, Matabeleland South 88%).

#### Referral for further management

Data from the 60 health facilities assessed showed that 48 health facilities did not refer a child for the management of malnutrition to either the OTP or the SC programme in 2019. Four health facilities were referring all children identified with malnutrition to the OTP even if they were eligible for referral to the SC programme. Only eight facilities (13%) were accurately referring children for the management of malnutrition to both the stabilisation centre and the outpatient programme.

### Outcome

The recovery rate in both the OTP and SC programme was low, with 58% and 57% of admitted children being eligible for discharge, respectively. A large proportion of children eligible for discharge (82% OTP, 75% SC programme) were not being discharged. Four percent of the children in both the SC programme and OTP died while on treatment. The Manicaland province had higher numbers of children missed but who were eligible for admission into both the OTP and SC programmes in comparison with the Matabeleland South province in 2019. The two provinces had a similar proportion (12%) of children who died while on treatment for malnutrition.

## Discussion

This study has found important gaps in the CGM programme in Zimbabwe. A high proportion of health facilities use dysfunctional equipment that can result in invalid weight and height measurements. In an evaluation that utilised the Monte Carlo simulation on anthropometric assessments carried out for children presenting with malnutrition, it was observed that random errors were very high when measuring tools were of insufficient quality, and the errors identified increased in magnitude when a bigger sample size was used.^24^ The utilisation of dysfunctional measuring tools reduces the reliability of individual anthropometric data and is likely to result in either an increase or a decrease in the prevalence of malnutrition in a setting.

The effect of training on the knowledge of health workers was mostly observed among the nurses who accurately responded to all aspects of the CGM programme assessed in this evaluation. Village health workers’ knowledge on the processes and function of weight-for-age monitoring was high, while that for MUAC and height-for-age monitoring was very low despite the evidence of training on CGM. The low knowledge levels can be attributed to the limited practice as a very few VHWs report having height boards. A literature review of evaluations of the CGM recommends continuous training, support and supervision for effective programme implementation.^20^ While training can assist in improving the output of VHWs, continued practice together with supportive supervision from health facility nurses can improve their understanding of height-for-age monitoring. In Mali, it was reported that training of community health workers on anthropometric measurements did not improve the quality of data collected; however, continuous supervision and additional training resulted in correct measurements.^25^

While a majority of nurses and VHWs had attended training on CGM, few nurses had been trained in the management of malnutrition and childhood illnesses. This gap in training increases the likelihood of missed opportunities for the management of malnutrition. While the CGM programme can be seen as a screening tool, a report by Mangasaryan et al.^26^ advocates for use of the programme as a diagnostic tool that can be used in timely identification of growth faltering in children, thereby preventing adverse nutrition outcomes and, at the same time, identifying children with malnutrition for prompt access to appropriate treatment.

We found a generally low uptake (< 50%) of the growth monitoring programme in Zimbabwe in the two provinces evaluated. A literature review of studies focusing on the CGM programme concluded that there was a general low uptake of the programme globally.^20^ When combined with other nutrition interventions, CGM such as community nutrition supplementation can result in reduced childhood mortality and reversing adverse nutrition outcomes, even with low levels of uptake.^26^ The CGM programme provides caregivers with an opportunity to have regular contact with health facilities, which is crucial for nutrition counselling and monitoring childhood illnesses.^20^ The missed opportunities for interaction between caregivers and health workers include the opportunity to discuss child nutrition, child growth and positive reinforcement of good caregiver practices, as well as good child growth.^20^

There is agreement from several reviews that growth monitoring on its own does not result in any positive change in children’s nutrition status.^20^ For the CGM programme to be effective, it should be accompanied by community-based health and nutrition interventions. Our study confirmed link between the CGM programme and both health facility and community-based nutrition and child health programmes. A high proportion of children (83%) identified as having adverse nutrition outcomes were not being referred to the IMAM programme at health facilities. A similar finding was reported in a study conducted in Malta that reported only 2% of eligible children were being referred for the treatment of malnutrition.^27^ A UN report on Zimbabwe reported that a majority of children identified as malnourished were being enrolled in community-based child feeding programmes.^28^ The role of food supplementation in child nutrition outcomes has mixed results, with most studies reporting that it may improve weight but has a limited impact on a child’s height-for-age measure.^29,30^ Children with severe forms of malnutrition received limited benefit from community-based feeding programmes.^16,17^

In a majority of the observations made in this evaluation, nurses explained the outcome of the growth monitoring process to the caregivers. Evidence has shown that appropriate counselling can result in growth catch-up among children with malnutrition.^31^ Caregiver understanding of the weight-for-age growth was found to be very high, while that for height for age was generally lacking. The finding is not surprising as VHWs had low understanding of the height-for-age indicator. Therefore, efforts need to be placed in strengthening community health cadres’ knowledge on the indicator and community health education activities that seek to improve understanding of the role of monitoring height for age. While health workers may often measure children’s weight and height, they rarely plotted this on the child health cards. The counselling sessions that they then hold with caregivers are thus impaired as parents do not visualise the growth of their children.

## Limitations

The evaluation was conducted in two provinces of Zimbabwe selected for being the least and most affected by childhood stunting. The two provinces are predominantly rural and may not be representative of the Zimbabwean population. Thus, results cannot be generalised for the urban population in Zimbabwe. The non-probability sampling method used in exit interviews also reduced the ability of the results to represent the Zimbabwean population. Observations of healthcare services may be affected by observation bias.

Finally, while the research had both interviews and secondary data analysis, information on the management of malnutrition was based mostly on secondary data analysis. The use of secondary data may not effectively result in understanding the possible reasons for a high proportion of children who were not referred for the management of either moderate or severe malnutrition. Thus, additional studies are required to ascertain the reasons why a high number of children missed during growth monitoring, and why a high number of children are not being admitted into the management of moderate and severe malnutrition while understanding why a large number of admitted children are not being discharged once enrolled.

## Conclusion and recommendations

A functional growth monitoring programme can assist in estimating child growth patterns and can provide real-time data to policy-makers on the adverse nutrition outcomes affecting children from the village level to the national level. The utility of the CGM programme in Zimbabwe as a diagnostic tool for malnutrition is compromised because of sub-optimal equipment. While a majority of the health workers and VHWs reported that they had been trained in growth monitoring, there is a need for further training in other services that support the programme. Referral of children eligible for malnutrition services was very low, and this could be improved if nurses are trained on how to identify children eligible for referral. Once the programme is strengthened, children with growth faltering will be linked to curative and preventive programmes. While there are efforts made by healthcare workers to reach children living in remote areas, much can be done to improve the current programme in order to reduce adverse nutrition outcomes, such as pre-school-based growth monitoring and home-based growth monitoring. For health facility-based growth monitoring to help in eliminating childhood malnutrition, there is a need for community growth monitoring and promotion activities to be strengthened. Regular supportive supervision visits by nutritionists while ensuring regular calibration of anthropometric tools can help to improve the quality of data collected. Implementation of social behaviour change activities that seek to improve the caregiver knowledge on the importance of height-for-age monitoring and other CGM priority areas can improve child nutrition outcomes. Future studies could do well to assess whether the CGM programme in Zimbabwe is reaching most children who are at risk of malnutrition.
